# Surgery

**Published:** 1858-05

**Authors:** 


### SURGERY.

1. CASE OF IMPERFORATE ANUS. PROPOSED MODIFICATION IN OPERATIVE INTERFERENCE. By *Redfern Davies, M.R.C.S.*, Birmingham.—The surgical epoch in which congenital imperforate anus will cease to be regarded as an anatomical *lusus naturæ*, or as an opprobrium to the art and science of our profession, will arrive only when success shall uniformly crown our efforts. At present, how few, how extremely rare, are any recorded cases of individuals born with this deformity, and living! The pages of our own and of foreign journals, though occasionally relating some solitary instance of partial success, are wonderfully barren in any accurate details on the subject; indeed, whether as to anatomy, physiology, or treatment, since the writings of Amussat, nothing has been improved upon, little or nothing has been in fact done.

The rectum, and its terminal apparatus, the anus, is formed, as is well known, by the descent of the former in the pelvis, which in due time meets the anus and becomes incorporated with it; while simultaneously the membraneous septum, which before had closed the end of the gut, becomes absorbed, giving place to what will best be fitted for the performance of the coming functions of life. In either of these parts of the economy, or both at the same time, arrest of development may occur. It may be complete or partial—thus producing the various forms of imperforate anus which have been seen. Malformations are also due to disease in intra-uterine life. Cases are reported showing evidence of inflammation having existed within the rectum, (*rectitis*,) whereby the coats became adherent and the rectum impervious.

*Anatomical characters of Imperforate Anus.*

1st, and simplest form of arrested development, is when the anus and rectum are both normally formed, but the membraneous septum of fœtal life still exists between the two.

2d. *Malformations of the anus.*—Of these there are an almost infinite variety. The integument of the perineum may pass over the mucous membrane of the aperture from one side to the other, or only partially over it—producing more or less blocking up of the passage, and usually presenting a kind of tubercle on the surface through which passes the fæces.

The sphincter may or may not be present.

3d. *Malformations in the rectum.*—When the rectum does not descend low enough in the pelvis to meet with the anus, but terminates in some form of *cul de sac* at a variable distance from it.

When the rectum opens into a part of the surrounding viscera, viz. bladder, urethra, or vagina.

4th. Every possible variety of the preceding classes existing together.

Case. *Past History.*—The mother of the child, a healthy primipara of twenty-eight years of age, was delivered of it at the full time, on December 19th, 1857. The child seemed, to all appearance, at birth, well and flourishing; but on the third day, no meconium having come away, the medical attendant elicited, in answer to his inquiries, that there existed some malformation about the child's bottom; which the nurse explained not having seen before owing to her bad sight. An imperforate anus was at once found to exist.

On being summoned in consultation, December 22d, by my friend, Dr. Cornelius Suckling, under whose care the child was, the following appearances presented:—

*Present State.*—A well-formed male child; seems weakly, but is tranquil. Urine quite clear, and made freely. On examining the perineum, no anal aperture is seen; the skin passes continuously from side to side; median raphé very marked and distinct. In the site of the anus the integument puts on a different color to adjacent parts; it is darker and has a peculiar appearance, simulating the usual anal wrinkles. On examining with the finger, and at the same time pressing upon the abdomen, or when the child cries, it is thought that in the direction of the rectum a fluctuation can be made out; the sensation is, however, by no means defined or certain. In all other directions an elastic, firm, resisting structure is evident. The bony outlines of the pelvis natural. The abdomen is bulging, hard, and tympanitic on percussion.

*Diagnosis.*—Absence of anus and of a portion of the rectum, probably from one to two inches. (Rectum does not open into viscera.)

*Operation.*—An incision—which, upon measurement, was found to be five-eighths of an inch—was commenced in the central line of the perineum, down to the coccyx, (in the part alluded to as marking the site of the anus.) Introducing the little finger, the sensation of the end of the rectum, though very distant, is now certain. Guided by that sensation, by the curve of the sacrum, and by the tuberosities of the ischium, the incision was carried by very gentle progression, cutting fibre by fibre, to a distance of two inches and a quarter, as was ascertained by actual measurement both at the time and afterwards. The extremity of the gut being satisfactorily recognized, an incision was made into it, and a grooved director pushed into the gut. An immediate escape of gas, which was very perceptible both to the ears and noses of the bystanders, as also by the welling out by the side of the director of meconium, announced that the rectum was opened. There was but very little loss of blood, estimated by the nurse as under a teaspoonful. A dose of castor-oil administered directly; a small oiled tent introduced into the wound; child put to bed.

*Vespere*, 10 p.m.—No more meconium has passed. Child seems easy.

*December* 23d, 10 a.m.—No meconium passed; child refuses the breast, seems fretful and ailing. On withdrawing the tent and introducing the finger into the wound, a few coagula are found at the top, closing the opening into the gut; on their being removed, a fresh escape of flatus and meconium, which now passes easily through the wound. He was ordered a purge of jalap.

*Vespere, same day.*—Child seems easy, but refuses nourishment; meconium comes away; urine clear.

*December* 24th.—Meconium and fæces come away easily. Child refuses nourishment.

*Vespere.*—About the same.

*December* 25th.—Died about noon.

*Post-mortem.*—Body of a dwindled appearance. Upon opening the cavity of the abdomen, no signs of peritoneal or inflammatory mischief could be anywhere traced; the contents appeared quite healthy, and the anatomical relations of the viscera as usual, save that there is a deficiency in the rectum, which is found to terminate in an infundibuliform *cul de sac* about the middle of the sacrum. Upon opening it, it is found to contain healthy fæces, and presents, about one-fourth of an inch posterior to the *cul de sac*, on its lower surface, an aperture three-eighths of an inch in diameter, through which a probe being introduced, passes out through the artificial anus in the perineum. The bladder is found quite natural. The rectum, as before said, terminated in a *cul de sac* of an infundibuliform shape, from which is prolonged, for about three-fourths of an inch, a fibrous cord. No muscular fibres could be found corresponding to the anus, though looked for, even by the microscope.

*Remarks.*—In the *Archives Générales* for the months of May, June, and July, in the past year, is a translation of an essay by Dr. Herman Friedberg, (*agrégé* to the Berlin Faculty of Medicine,) on Artificial Anus. The subject is discussed at length,—it is the most complete *resumé* of cases to be met with in any language; and, from the high character of the author of the paper, much weight is attached to his doctrines and practice. After commenting upon the advantage of an opening in the perinæum, as opposed to one in any other site, M. H. Friedberg goes on to say, that simple opening of the rectum through the perineum ought, in the present day, to be completely abandoned. "I do not know," he says, "a single case in which an opening by a trochar has preserved life permanently, when the closed extremity of the rectum has been distant from the normal site of the anus;" and, furthermore, he cites in corroboration of his opinion the words of M. Roser, who "has not found any detailed account, not even one, which tends to show that any decided improvement has been obtained by the employment of the trochar, when the rectum has been absent a distance of one inch." The operation that he strongly contends for is that of Amussat, viz. the bringing down the gut when an opening has been made in it, and stitching it to the outlet in the perineum. In support of this, he adduces two cases in which the rectum was distant one inch and a half and three inches respectively, (they, however, died, the one in six, and the other in nine months;) together with the four successes of Amussat, which, as Erichsen observes, singularly all occurred at Brest. Certainly, the Professor does not prove much by numbers. The advantages that he claims for it are beyond doubt good and substantial, viz. that there is a mucous membrane lining the whole tract of the canal; that the evacuations are more easily accomplished; that the natural tendency in canals not so provided to gradually contract, and finally become completely closed, is prevented; that the irritation and danger arising from the contact of effete matter with tissues not intended for such contact is also obviated. Against so formidable an array of arguments in favor of this line of conduct, the reasons that induced me, at the time when the rectum was opened, to forego even the attempt to bring it down, were, that I deemed the distance, two and a quarter inches, at which it was situated from the external opening, to be so great as to preclude the possibility of so doing. Bound down as the rectum is by its fold of peritoneum, the mesorectum, I feared to encounter the almost certain dangers of peritonitis, or pelvic cellulitis, which must inevitably be the probable consequence of the laceration of its connective tissue, to permit of its descent for such a distance. Besides, at such a depth, how great an uncertainty there must be as to what the forceps might seize hold of. A free outlet having been established, the exigencies of life were provided for, and the vital functions were, for the time, fully as well performed as they could have been even had the gut been brought down. So far the operation consisted in one of simple puncture; and had it been intended for it to remain as such, the somewhat violent and dogmatic fiats of the two German *savans*, quoted a few sentences back, would be, as I shall directly show, not absolutely justifiable. One case by Schleiss (in 1853) is narrated as successful; and up to the present hour its result has not been impugned. Another, where the rectum was absent about two and a half inches, I had the opportunity of witnessing while in Paris last year; the case was under the care of M. Maisonneuve, and presented to the Academie de Chirurgie, also reported in the *Gazette des Hôpitaux,—vide* Nos. 22 and 53, 1857. There is at present—aged some forty years—a gentleman in this town who was so operated upon, and who walks about, a living monument of its success. The fact, therefore, that in certain isolated cases a fortunate issue results, is certain; and it is also as certain that they are very, very much the exception to an all but universal law, that children born with imperforate anus die. To what, then, shall we attribute this result? The child is born, to all appearance, healthy, save this defect; which only kills by its mechanically preventing the exit of effete matter, and its attendant consequences. It would, therefore, appear that to overcome this difficulty would be to solve the question; and doubtless such will be the result when the operative procédé shall be improved, and so in proportion will lives be saved, save in those cases where the vital powers of the child are below par (as in part evidenced by the congenital deformity) and of themselves unequal to carry on life, much less when this embarrassment is added to them.

With all due deference, therefore, to the opinions of others, and in the hopes that it will receive whatever of attention it may merit at their hands, I beg to lay before the opinion of my more experienced professional brethren the following modification in the operative interference usually adopted in these cases, which I had intended, had the patient survived a sufficient length of time, to carry into effect. As far as can be judged by the evidence of the published cases, death is the consequence of different causes, according as the rectum is, or is not, brought to the opening of the wound. If it is, death ensues from the injuries inflicted by so doing. If it is not, death ensues, but secondarily, in consequence of the difficulty to defecation being only partially removed. I would propose, therefore, to combine these two procédés, and endeavor to obtain, by extending the operative measures over a considerable time, immunity from the evils of both: viz. supposing, in the first instance, that an opening had been made (as was done) into the rectum, nature being relieved, had not other influences intervened, the child would have lived *pro tem.;* but then comes into consideration the subsequent difficulty in passing the stool, owing to a gradually narrowing of the passage. All this is said to be due to the mucous membrane not being continuous with the outlet.

To remedy this, therefore, when the parts have recovered from the effects of the first operation, introduce a pair of forceps, and, seizing hold of the lips of the opening into the rectum, endeavor to bring it down, not by one vigorous and decisive holding on by the forceps, and by main force bringing the gut to the external orifice, but by gently and repeatedly soliciting its descent, introducing the forceps at certain intervals, and gradually endeavoring to accomplish the end. If the rectum can be so moved from its position, and be brought lower down in the pelvis (and so by repeated attempts it has been proved) by one forcible extension, and even that sometimes crowned by success, how much more likely is it that success should attend the proceeding when, by the almost imperceptible tractions made upon it, the great causes of failure, viz. peritonitis and pelvic cellulitis, would be removed, owing to the small amount of disturbance that would take place in the soft parts. Although, as far as I am aware, this procédé by successive stages has never before been broached in any writings on the subject, the idea was taken from a case reported in the *Lancet*, vol. i. p. 493, 1846, in which an incision was made into the perineum for a distance of three inches, and on the second day an attempt was made, by gently pulling, to draw down the gut, which was not, however, fastened to the external opening. One month afterwards the child was doing well.

I am fully aware that there is a vast deal of essential difference between this procédé and the one I advocate; nevertheless, accomplishing the end by successive stages, is in this case shadowed out, and will, I trust, assume a definite status in surgery."—*Edin. Med. Journ.*, March, 1858.

2. LARGE POPLITEAL ANEURISM TREATED BY PRESSURE, AND AFTERWARDS BY LIGATURE.—An interesting case of aneurism is now under Mr. Birkett's care at Guy's Hospital, which exemplifies a class in which the compression-plan often fails. For success, that method evidently requires a certain amount of vigor on the part of the patient, and of good coagulating power on the part of the blood. We have had to record many instances of failure in feeble, poor-blooded persons, and the following adds another to the list:—A laboring man, giving his age as thirty-six, but looking much older, was admitted with a very large popliteal aneurism in the right ham. He stated that he had continued at work until the previous day, and did not think that the tumor had existed for more than six weeks. The contents of the sac appeared to be wholly fluid blood. The pressure-treatment was commenced, and persevered in with some interruptions, for twelve days, but without producing any appreciable alteration in the contents of the sac. The integuments on the outer side of the tumor had, however, become discolored, and Mr. Birkett was apprehensive that ulceration of the sac was impending. It was accordingly determined not to delay longer the placing of a ligature on the femoral artery. This was done at the usual site. The ligature came away on the ninth day, and the wound healed well. It is to be noticed that the diminution in bulk of the tumor appeared to be solely by absorption of fluid blood, and not by any process of solidification. The sac, indeed, although much smaller, is still soft, and contains much fluid blood. The man's general health has much improved since the operation.—*Medical Times and Gazette*, Jan. 30th, 1858.

3. MALIGNANT TUMOR NEAR THE BREAST AT AN UNUSUALLY EARLY AGE.—When malignant growths occur to persons young in years, it is well known that they are usually either of the medullary or melanotic form, very rarely scirrhous, and with still greater rarity epithelial. In some instances, however, and especially in or near the mammary gland, a form of very dense medullary is met with which approaches closely to the character of scirrhus. Of this the following is an example:—A delicate, pale-faced girl, aged twenty-three, unmarried, was admitted into St. Bartholomew's, under Mr. Lawrence's care, with a tumor the size of an egg situated in the outer and upper part of the left breast. The skin over it was ulcerated in several parts, and was tense, thin, and shining. Hemorrhage had several times occurred from the ulcerated spots. None of the axillary glands could be ascertained to be enlarged. There was no history of hereditary tendency to malignant diseases. She attributed the tumor to a blow she had received on the part about a year previously. Mr. Lawrence excised the mass, which was found not to be connected with the mammary gland itself. On making a section of it, its lower part was found of great firmness, almost as much so as if true scirrhus, while in its upper were several cysts, containing a bloody fluid. The wound slowly healed, and the woman's recovery was somewhat tedious.—*Ibid.*

4. EXCISION OF THE ANKLE-JOINT.—This operation was performed last week by Mr. Hussey, at the Radcliffe Infirmary, Oxford. The patient was a countryman, aged twenty-six, suffering from disease of the ankle-joint, the origin of which he attributed to a sprain. The malleoli, with the ends of the tibia and fibula, were removed with cutting pliers, and the articular surface of the astragalus was cut away with a gouge. No tendon was divided, and no vessel needed tying.—*Ibid.*

5. COLLODION AND CASTOR OIL AS AN ARTIFICIAL CUTICLE.—The mixture has been used of late with success in King's College Hospital, as an application to burns and abrasions, to form a sort of artificial cuticle. It has been used at the suggestion of Dr. Savage, at the Samaritan Hospital, in two cases of vesico-vaginal fistula, now there under the care of Mr. Spencer Wells. In one of these cases there is a recto-vaginal fistula also. In both the excoriation of the labia, perinæum, and thighs, from the constant dribbling of urine and the consequent smarting, has been very distressing. Extreme cleanliness, careful drying of the parts, and the use of simple ointment, afforded but little relief. The mixture of one part of collodion to two parts of castor oil was therefore used, and gave the most marked relief. It causes some smarting for a few minutes after its application, but it then forms a smooth, elastic coating or varnish, which resists the action of the urine for many hours, and effectually protects the excoriated skin from the irritating fluid.—*Ibid.*

